# Modified mattress suture

**DOI:** 10.1308/003588412X13373405385214k

**Published:** 2012-07

**Authors:** S Goudie, S Dreyer, R Siddiqi

**Affiliations:** NHS Borders,UK

**Figure 1:**
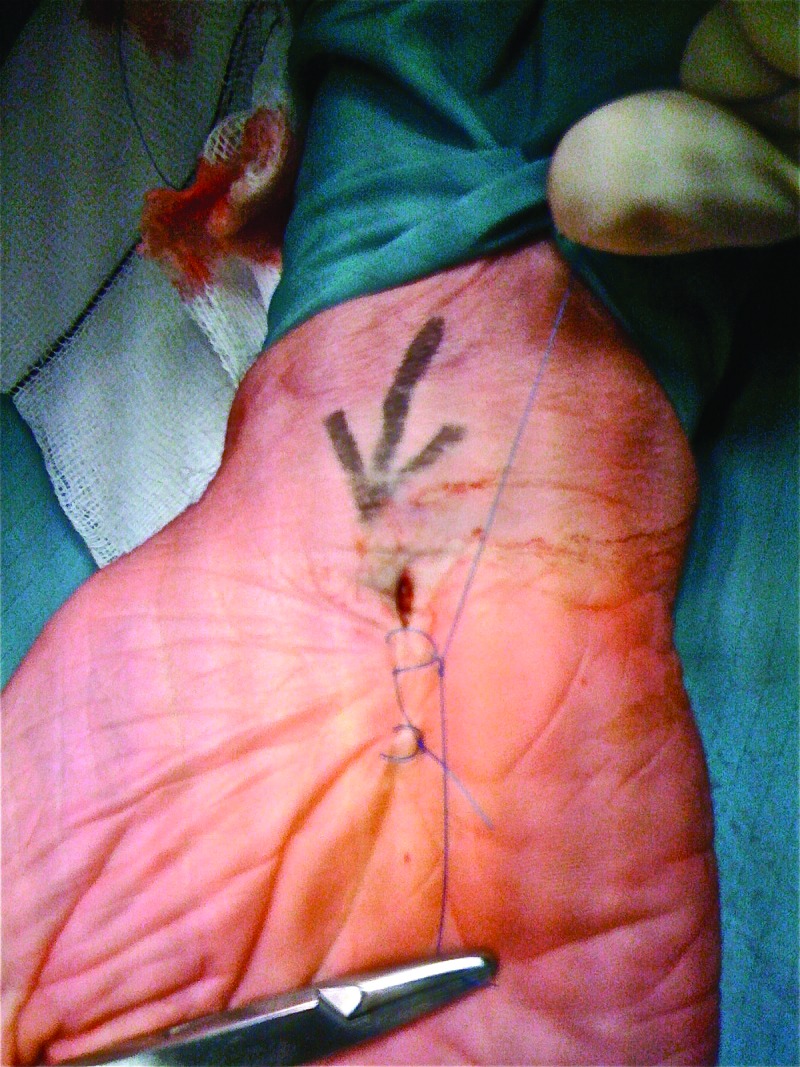
Modified mattress suture

Traditional mattress sutures prove difficult to remove when the knot becomes buried in swollen tissue. We describe a suture with the benefits of a mattress suture that can be removed more easily:

1. Starting on the ‘near-side’, cross the wound twice as per a regular horizontal mattress suture, leaving the loop proud.
2. Pass one free end through the loop and pull to the near-side of the wound.
3. Tie on the near-side, ensuring suture material of the loop bridges the outside of the wound.
4. This will remain easily accessible for removal regardless of swelling (Fig 1).

